# Ontology based autonomous robot task processing framework

**DOI:** 10.3389/fnbot.2024.1401075

**Published:** 2024-05-07

**Authors:** Yueguang Ge, Shaolin Zhang, Yinghao Cai, Tao Lu, Haitao Wang, Xiaolong Hui, Shuo Wang

**Affiliations:** ^1^The State Key Laboratory of Multimodal Artificial Intelligence Systems, Institute of Automation, Chinese Academy of Sciences, Beijing, China; ^2^The School of Artificial Intelligence, University of Chinese Academy of Sciences, Beijing, China; ^3^The Center for Excellence in Brain Science and Intelligence Technology, Chinese Academy of Sciences, Shanghai, China

**Keywords:** service robot, knowledge-enabled robot, ontology, knowledge representation, task planning

## Abstract

**Introduction:**

In recent years, the perceptual capabilities of robots have been significantly enhanced. However, the task execution of the robots still lacks adaptive capabilities in unstructured and dynamic environments.

**Methods:**

In this paper, we propose an ontology based autonomous robot task processing framework (ARTProF), to improve the robot's adaptability within unstructured and dynamic environments. ARTProF unifies ontological knowledge representation, reasoning, and autonomous task planning and execution into a single framework. The interface between the knowledge base and neural network-based object detection is first introduced in ARTProF to improve the robot's perception capabilities. A knowledge-driven manipulation operator based on Robot Operating System (ROS) is then designed to facilitate the interaction between the knowledge base and the robot's primitive actions. Additionally, an operation similarity model is proposed to endow the robot with the ability to generalize to novel objects. Finally, a dynamic task planning algorithm, leveraging ontological knowledge, equips the robot with adaptability to execute tasks in unstructured and dynamic environments.

**Results:**

Experimental results on real-world scenarios and simulations demonstrate the effectiveness and efficiency of the proposed ARTProF framework.

**Discussion:**

In future work, we will focus on refining the ARTProF framework by integrating neurosymbolic inference.

## 1 Introduction

Benefiting from the rapid advancements in artificial intelligence and robotics, the perception capabilities of robots have been significantly improved in recent years. Robots are now able to accomplish basic tasks such as object recognition, navigation, and manipulation. However, the task execution of the robots still lacks adaptive capabilities in unstructured and dynamic environments. Consider the basic task of retrieving apples, if the robot can visually perceive the apple, the robot is able to successfully grasp it and execute the corresponding action. However, if the robot is in an indoor environment and the apple is placed in a box or drawer, the robot lacks the ability to reason about the task. The lack of cognitive and reasoning ability poses a critical bottleneck for the robot to accomplish the task. Specifically, robots lack the fundamental understanding of commonsense knowledge. Their cognitive abilities remain confined to basic object recognition, limiting their capacity to tasks in unstructured and dynamic environments.

Symbolism believes that cognition is a form of symbolic processing in ontology, suggesting that human thought processes can always be described through specific symbols. Ontology, which can effectively describe the hierarchical structures and semantics of different concepts, has become an important tool for robot's reasoning capacities (Olivares-Alarcos et al., 2019; Paulius and Sun, 2019). Suh et al. (2007) proposed an ontology-based multi-level robot knowledge framework (OMRKF) , which achieves the semantic cognitive representation of robots by defining four knowledge types: perception, model, activity, and context. Ontological knowledge reasoning is achieved through defining knowledge axioms and rules, providing the ability to query semantic knowledge effectively. Tenorth et al. (2010), Tenorth and Beetz (2013), Tenorth and Beetz (2017) and Beetz et al. (2018) proposed an ontology-based knowledge processing system named KnowRob, which built a semantic framework integrating multi-source heterogeneous information. KnowRob has the capacity for both knowledge representation and reasoning. Leveraging ontology as the knowledge carrier enables the effective characterization of multiple and complex classes, attributes and relationship of knowledge. Based on the representation of classes and attributes, KnowRob can align knowledge with objects in the real scenario and generate a large number of instance descriptions through inheritance operations. Rule-based reasoning approaches empower the customization of rules tailored to specific application scenarios, thereby enabling user-defined reasoning processes. Based on KnowRob, Beetz et al. (2010) proposed CRAM cognitive framework (Beetz et al., 2010, 2023). This framework addresses the challenge of missing information in daily tasks by utilizing CRAM Plan Language (CPL) to build action plans. By leveraging knowledge reasoning, CRAM fills in the gaps in action plans, which enables robots to execute daily operations effectively. ORO (Lemaignan et al., 2010; Lemaignan, 2013) proposed a general knowledge representation framework for autonomous robot-human interaction processes. It aims to enhance the robot's interaction capabilities in complex human living environments. The tasks include object recognition, natural language interaction, task planning, and collaboration with other robots or humans. The knowledge in ORO is grounded on an upper-level ontology built on OpenCyc (Lenat, 1995), which allows for the addition of new ontologies on top of the upper-level ontology. ORO employs Pellet (Sirin et al., 2007) for ontology knowledge query and reasoning. Li et al. (2017) introduced the Smart and Networking Underwater Robots in Cooperation Meshes (SWARMs), which aims to address information heterogeneity and facilitate uniform comprehension among robots regarding exchanged information.

The aforementioned knowledge frameworks use ontology as the basis for knowledge representation and reasoning, which could provide rich semantic information for robots. OMRKF (Suh et al., 2007) addresses the low-level perception by storing SIFT visual features in a hierarchical symbolic architecture, making it difficult to extend to more complex entities or actions. KnowRob adopts an encyclopedia form to build the semantic knowledge model, which lacks the top-level design for tasks. CRAM focuses on completing task parameters through knowledge but lacks emphasis on dynamically generating action execution sequences in tasks. The ORO knowledge management system highlights the interaction between robots and humans. The ontology in SWARMs is specialized for unmanned underwater robots, limiting its applicability to other types of robot applications. Moreover, representative work of knowledge frameworks such as RoboEarth (Waibel et al., 2011), OPEN-EASE (Beetz et al., 2015), and RoboBrain (Saxena et al., 2014) emphasize more on knowledge sharing among different robots. They do not offer task processing tailored for robot manipulations in dynamic environments.

Recently, deep learning has achieved remarkable breakthroughs in vision tasks such as object detection and recognition. R-CNN series from R-CNN to Mask R-CNN (Girshick, 2015; Ren et al., 2015; He et al., 2017; Bharati and Pramanik, 2020), YOLO series (Redmon et al., 2016; Redmon and Farhadi, 2017, 2018; Jiang et al., 2022), and SSD (Liu et al., 2016; Zhai et al., 2020) are representative works in deep learning-based object detection. Deep learning-based approaches have greatly improved the performance of object detection and recognition compared with manually designed features. Meanwhile, Robot Operating System (ROS), as a communication framework specifically designed for robot software development, has attracted much attention. ROS hosts a varieties of algorithms such as Gmapping for laser-based SLAM (Simultaneous Localization and Mapping) and MoveIt for robotic arm motion planning. In a semantic knowledge-assisted robot, beyond achieving the dynamic update of the knowledge base by combining robot perception system with the knowledge-driven decision-making control, it is also necessary to address the easy deployment of new perception and control algorithms. To this end, this paper proposes an ontology autonomous robot task processing framework (ARTProF). This framework seamlessly integrates knowledge representation, knowledge reasoning, and autonomous task planning and execution.

ARTProF is based on ontological knowledge representation and reasoning. In the proposed framework, the instances in the knowledge base are generated with neural network-based object detection algorithms. The proposed framework also defines ROS-based manipulation operators for the robot, which establishes the connections between the primitive actions of the robot and the objects it interacts with. The integration with the ROS system facilitates the relationship between the knowledge system and the robot. Moreover, the proposed framework includes an operation similarity model for different objects. When the robot is operating a novel object, the robot's action is selected autonomously according to the similarity model, which endows the robot with the ability to manipulate generalization. Moreover, ARTProF achieves dynamic task planning by leveraging knowledge reasoning. The robot can autonomously and dynamically organize action sequences to complete tasks in diverse environmental conditions. Compared with existing knowledge frameworks, ARTProF offers the following advantages: (1) ARTProF addresses the task demands in dynamic and uncertain environments by supporting the representation and reasoning of common sense and task knowledge, dynamic knowledge generation, task planning and execution. These functionalities provide a comprehensive support for robot task execution. (2) An operation similarity model is proposed to facilitate operation transfer among different objects. Objects with similar characteristics are manipulated in a similar manner. (3) A dynamic task planning algorithm is proposed based on the ARTProF framework. The generated plans satisfy the execution constraints defined in the prior knowledge during robot task execution.

The paper is organized as follows. Section 2 introduces the basic architecture, knowledge representation and reasoning of the ARTProF. Section 3 gives the design and implementation of the perception system, where the instances in the knowledge base are dynamically generated. Section 4 introduces the knowledge-guided manipulation operators. Section 5 introduces the dynamic task planning based on knowledge reasoning. Section 6 presents our experimental result and analysis on both real-world scenarios and simulations. Finally, we conclude the paper in Section 7.

## 2 The framework of ARTProF

**Figure 1:**
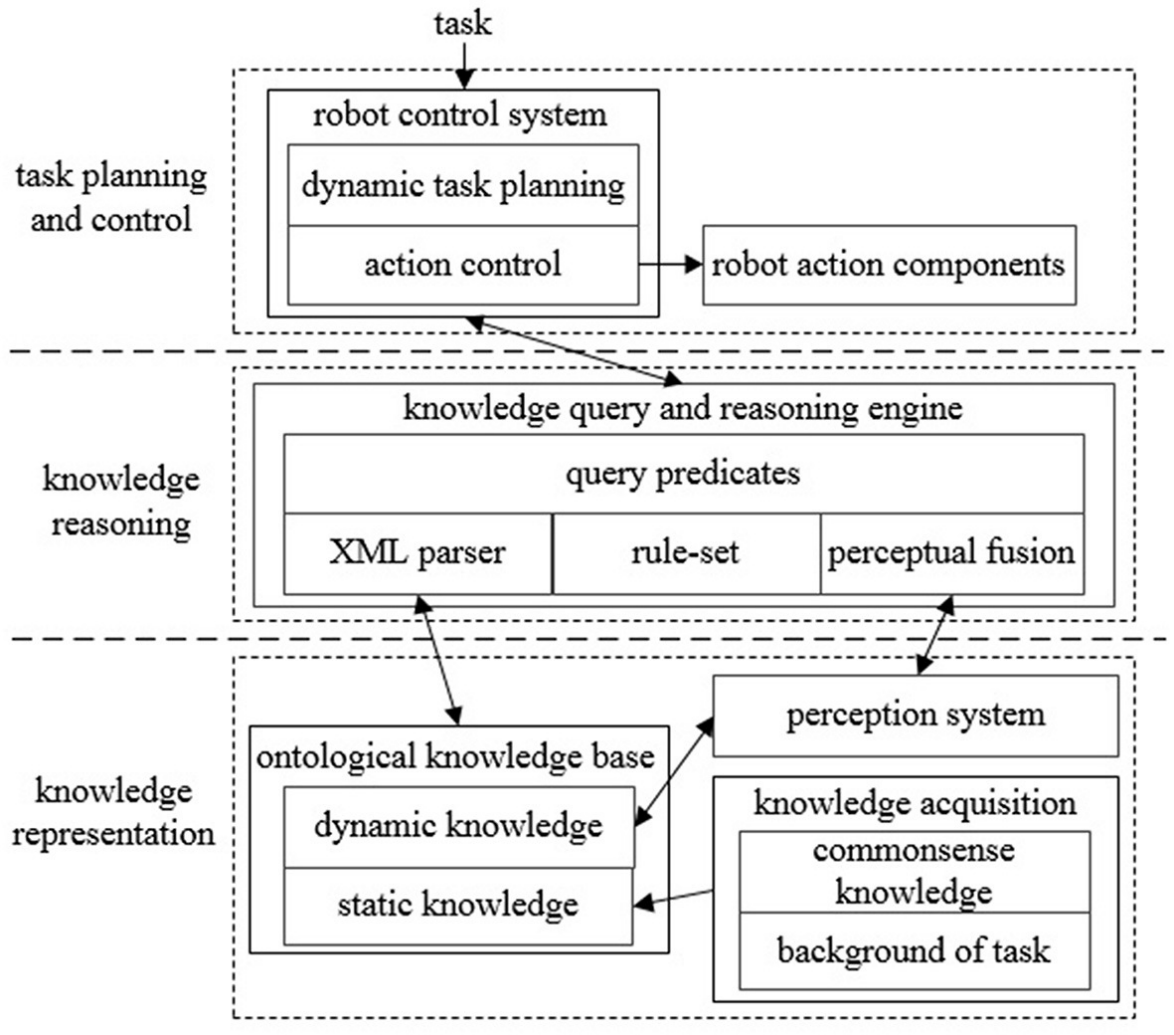
The architecture of ARTProF. The knowledge representation layer utilizes the OWL language to describe static and dynamic ontology knowledge related to the environment and tasks. The knowledge reasoning layer achieves ontology parsing and constructs inference rules grounded in logic programming. Additionally, it integrates perception algorithms to implement logical reasoning process. The task planning and control layer incorporates a knowledge-based dynamic planning algorithm to enable autonomous robot task execution.

As the knowledge processing and task execution system for autonomous robots, ARTProF is able to handle diverse knowledge types such as environment knowledge and task knowledge. ARTProF also presents capacities for flexible knowledge reasoning. Through integration of the perception and control systems, ARTProF achieves knowledge-based autonomous control, enabling the robot to execute everyday manipulation tasks. The framework of ARTProF is shown in Figure 1.

In ARTProF, the ontological knowledge base is constructed using the description logic (DL), which includes classes, attributes, and instances needed to describe the object. The ontological knowledge base is extensible, allowing the derivation of new classes from existing ones and incorporation of new object instances. The ontological knowledge is denoted as *D* = (*T, A*), where *T* is the TBox (Terminology Box) and *A* is the ABox (Assertional Box). The TBox represents static knowledge built from commonsense knowledge and task-specific background information. It defines concepts and relationship between concepts such as abstract classes, inherent attributes, and relationships between classes. On the contrary, the ABox represents dynamic knowledge derived from the real-time data acquired by the robot perception system. This dynamic knowledge represents specific events such as object instances, size, pose, and state. We use Web Ontology Language (OWL; Motik et al., 2009) to store the description logic knowledge in XML-based files. Originally developed for knowledge representation in the semantic web, OWL has now become a general knowledge representation format capable of describing all aspects of objects, actions, time events, attributes, and their relationships.

**Figure 2:**
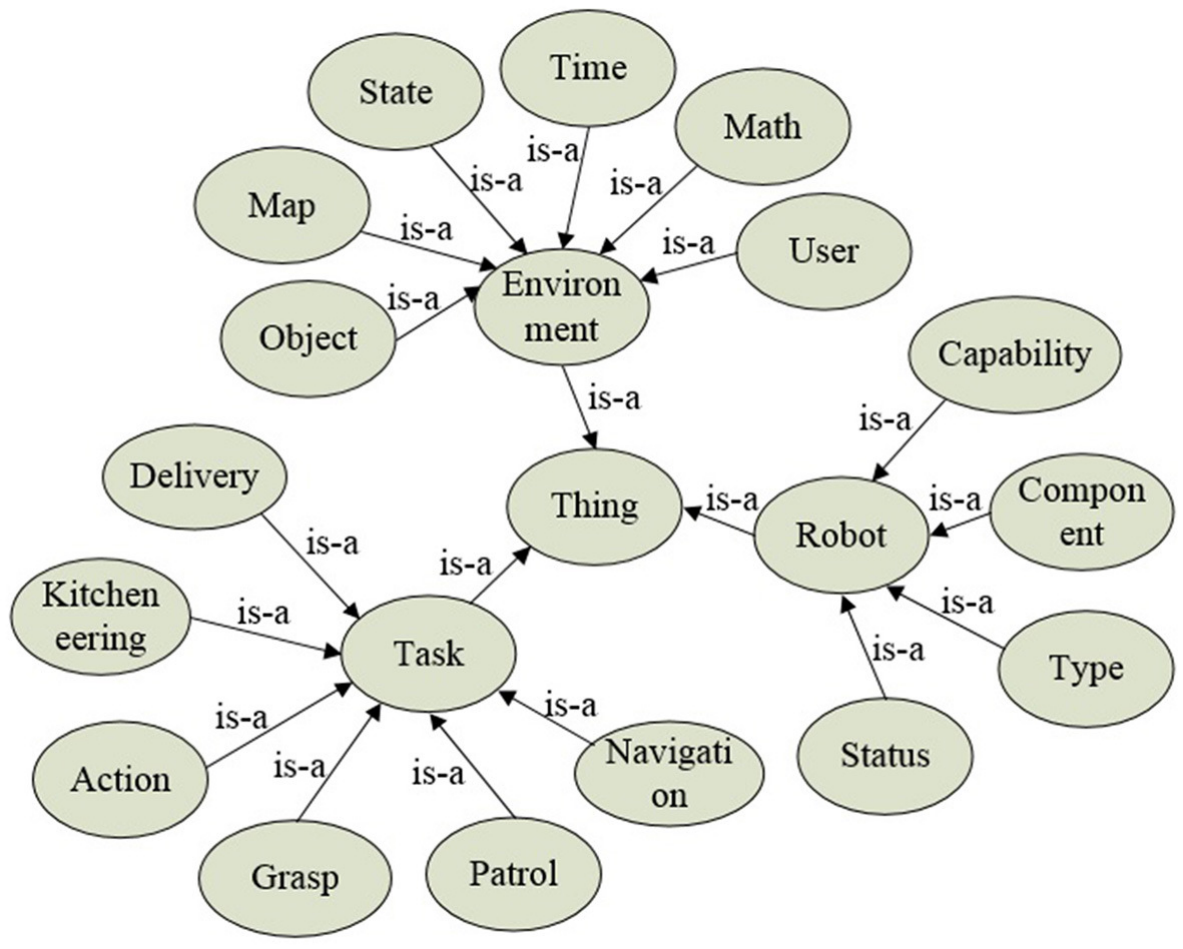
The semantic knowledge of robot task processing in ARTProF. The nodes and edges in the graph correspond to classes (instances) and properties in the OWL language, respectively.

The semantic knowledge of robot task processing is shown in Figure 2. The “Environment” class describes information related to the environment where the robot is located. It includes “Object” for the classes of the operated object and its associated semantic attributes, “State” for defining the object's state, “Map” for representing the semantic layout of the task environment, “Time” for temporal concepts, “Math” for mathematical models and algorithms, and “User” for robot user-related knowledge including identity and task-specific information such as usernames, habits, interests, etc. The “Task” class defines basic primitive actions necessary for the robot's task execution. Upon receiving a task, semantic understanding, action decomposition, and dynamic planning are facilitated through the “Task” class. The “Robot” class describes the attributes of the robot itself. It includes “Capability” for function descriptions, “Component” for hardware configuration, “Type” for the robot category (e.g., industrial and service), and “Status” for describing the robot's operational states (e.g., working, shutdown, and charging).

There are many inference tasks implemented by DL inference engines, such as Racer (Haarslev and Möller, 2001), Pellet (Sirin et al., 2007), and HermiT (Shearer et al., 2008), which are operated by maintaining a complete knowledge base in memory. This reasoning mechanism requires reasoning on the entire knowledge base whenever there are changes, which is both time-consuming and not suitable for the reasoning in dynamic environments. In ARTProF, we choose a purely memory-based infrastructure for efficiency. The knowledge query and reasoning engine of ARTProF utilizes the semweb library (Wielemaker et al., 2003) as an XML parser to convert the XML parse-tree from the OWL ontology file into a Prolog list of triples. We further employ the rule-based reasoning used in SWI-Prolog (Wielemaker et al., 2012). SWI-Prolog allows to customize the rules according to specific application scenarios, enabling reasoning through querying the predicates (Vassiliadis et al., 2009).

When the robot is interacting with objects, the robot needs to understand the object's attributes, infer the object's location, and formulate the manipulation policies. These decisions necessitate relating the abstracted knowledge about objects to the physical entities in the environment. Moreover, the knowledge base is required to associate with the robot perception system. To seamlessly integrate the perceived visual information into the knowledge query and reasoning processes, the perceptual fusion model is designed in ARTProF. This model synchronously transforms the perceived visual information into dynamic knowledge.

**Figure 3:**
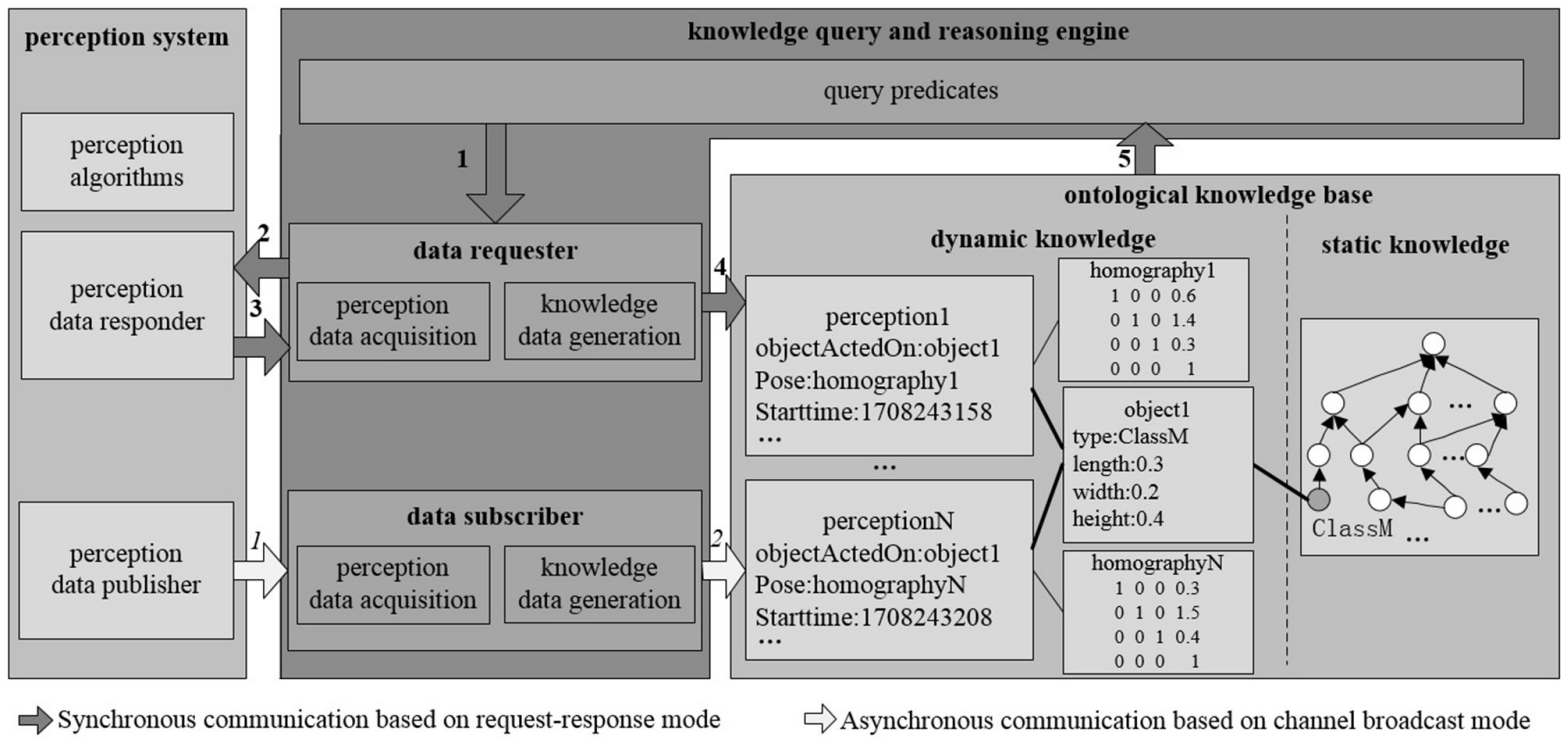
The interaction process between the knowledge base and the perception system in ARTProF. ARTProF provides two connection modes: synchronous communication (request-response mode) and asynchronous communication (channel broadcast mode).

Figure 3 illustrates the interaction between the knowledge base and the perception system within ARTProF. ARTProF provides two connection modes between the knowledge base and the robot perception system: (1) synchronous communication (request-response mode). This mode allows on-demand perception of objects while querying the knowledge base to generate object instances. It achieves synchronous updates of the knowledge base by incorporating the perceived object instances. (2) Asynchronous communication (channel broadcast mode). The knowledge base asynchronously updates by passively listening to the published object detection results. The generated object instances mainly include attributes such as ID, category, pose, size, material, etc. Since the pose of the object can be varied, an intermediate perception instance is added to bridge the object instance and its pose, capturing the object's state at a specific timestamp.

**Figure 4:**
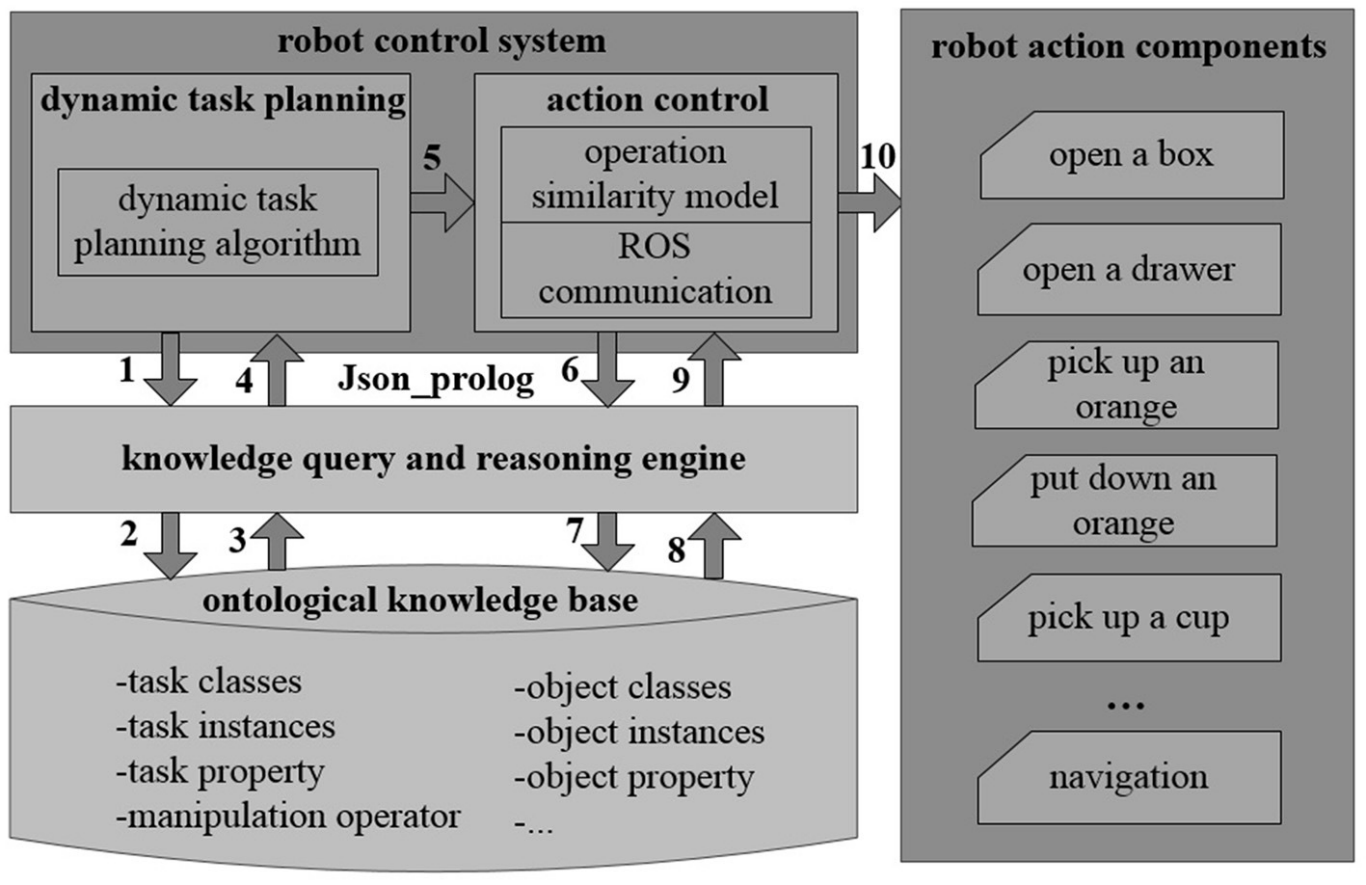
Knowledge-based control system in ARTProf. The robot control system transforms the control decision into a knowledge reasoning task. The robot control is obtained by querying the knowledge base, where different primitive actions of the robot are combined.

The robot control system transforms the control decision into a knowledge reasoning task. The robot control is obtained by querying the knowledge base, where different primitive actions of the robot are combined as illustrated in Figure 4. The dynamic task planning module employs a dynamic planning algorithm (Algorithm 1). Upon acquiring task definitions through knowledge base queries, it derives a sequence of primitive actions required for the task. The action control module also inquires the object characteristics through knowledge queries. It then determines suitable manipulation operators according to an operation similarity model. Subsequently, it executes the corresponding primitive actions leveraging the ROS communication mechanism. The interaction between the robot control system and the knowledge query and reasoning engine is realized through calling Json_prolog in high-level languages such as Python, C++, and Java.

**Algorithm 1:**
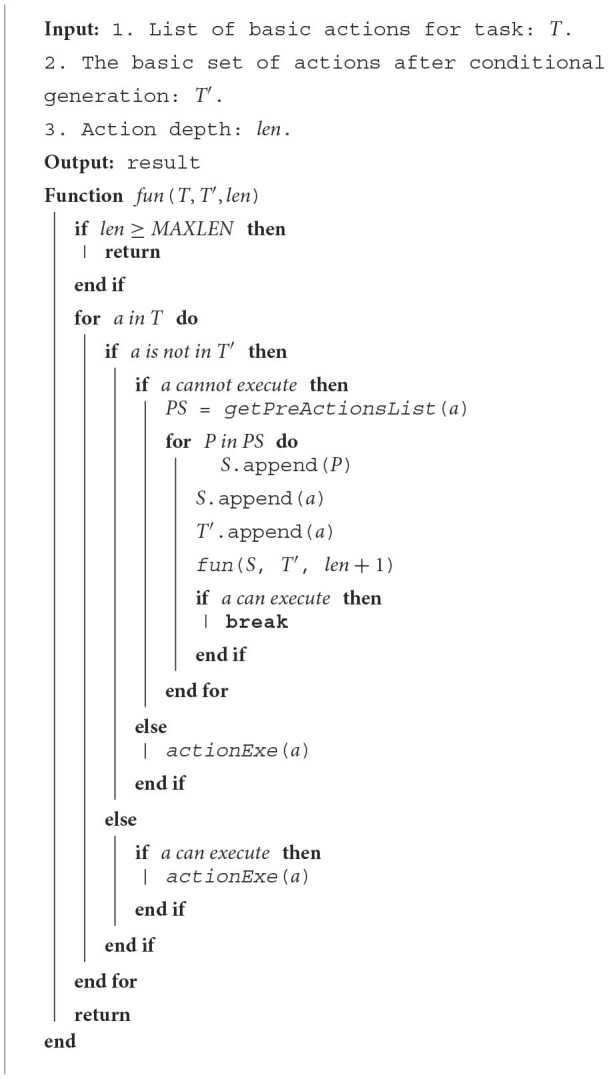
Dynamic task planning algorithm.

## 3 The perception system

In ARTProF, we present a unified communication interface between the knowledge base and the perception system. Recently, deep learning has revolutionized object detection. The performance of object detection has been improved significantly (Girshick, 2015; Ren et al., 2015; Redmon et al., 2016; He et al., 2017; Redmon and Farhadi, 2017, 2018; Bharati and Pramanik, 2020; Jiang et al., 2022). Moreover, the 6D pose of the detected objects can be obtained through PoseCNN (Xiang et al., 2018), PVNet (Peng et al., 2019), and SilhoNet (Billings and Johnson-Roberson, 2019), etc. By defining a unified communication interface between the knowledge base and neural network-based perception algorithms, detection and recognition of objects at different abstraction levels can be achieved.

**Figure 5:**
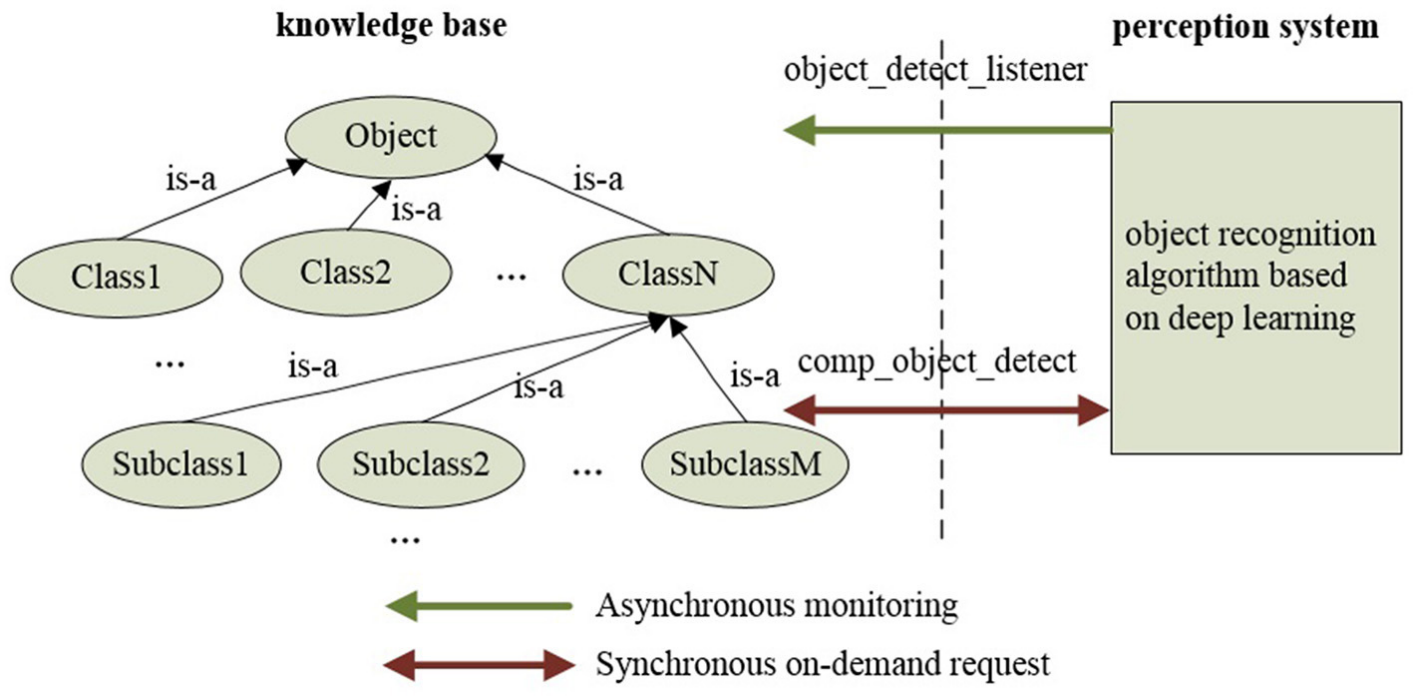
The communication modes between the knowledge base and the perception system in ARTProF: synchronous on-demand in a request-response manner and asynchronous in a passive listening manner.

Figure 5 illustrates the communication modes between the knowledge base and the perception system. The “object_detect_listener” and “comp_object_detect” are different communication modes: synchronous on-demand in a request-response manner (“comp_object_detect”) and asynchronous in a passive listening manner (“object_detect_listener”), respectively.

**Figure 6:**
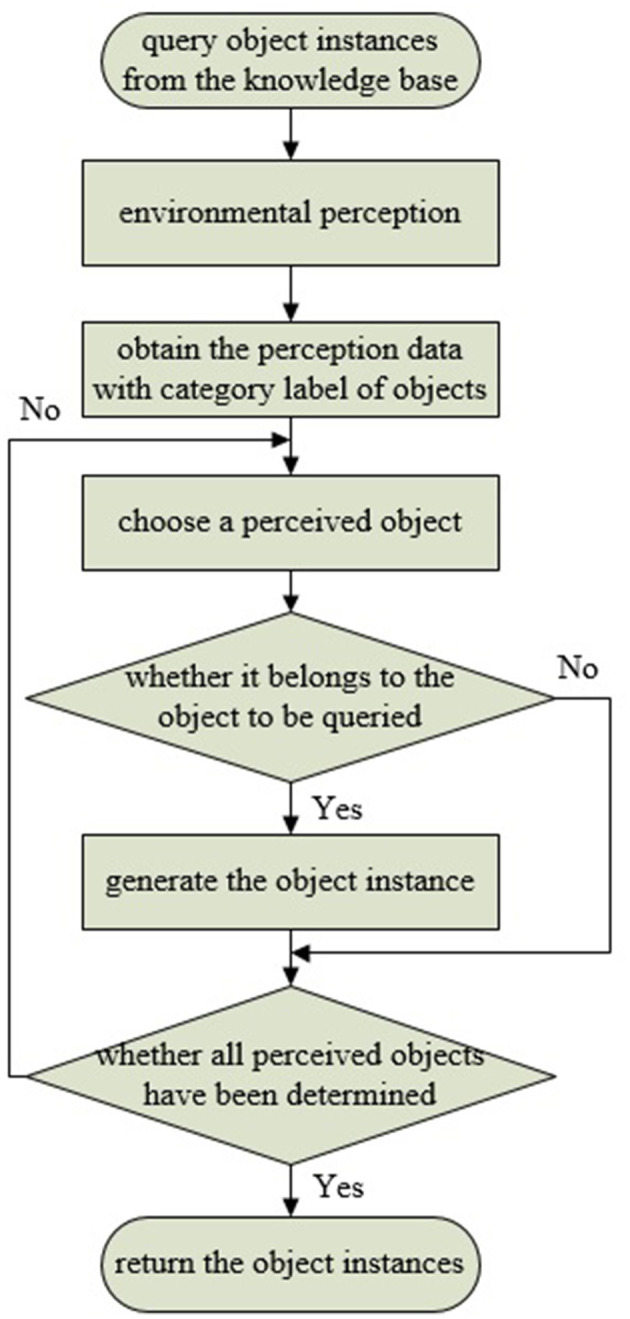
Synchronous communication. When querying a specific object through the “object detect listener” in the knowledge base, it triggers the perception system to acquire instances of the queried object in the environment through synchronous communication.

**Figure 7:**
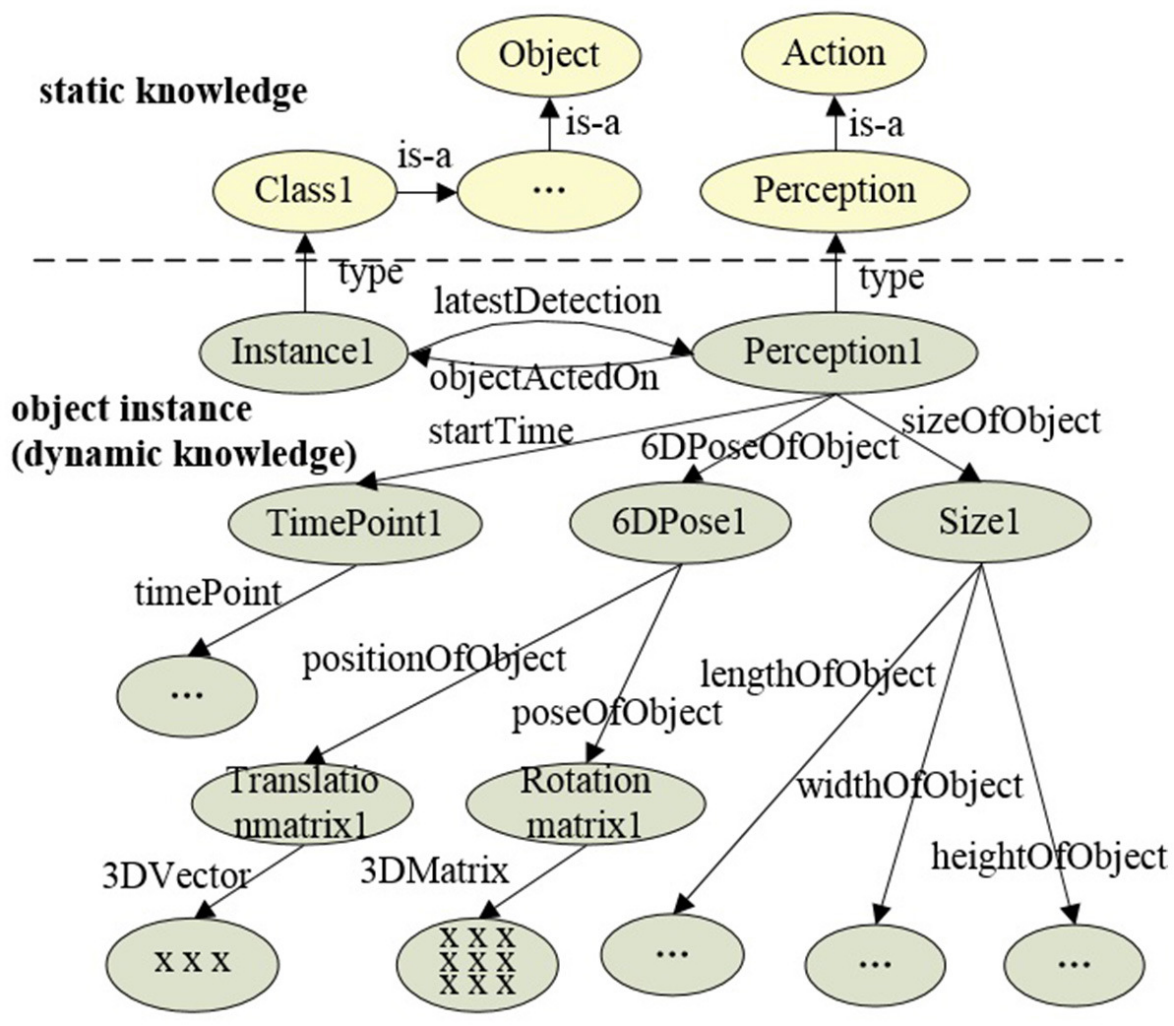
Knowledge topology of the “Instance1.” The nodes in the graph are represented by classes or instances in OWL, and the edges are represented by properties in OWL.

In the synchronous communication mode, the object perception algorithm is encapsulated as a ROS service node. The connection is triggered by a custom Prolog predicate, which implements ROS service invocation, accesses the knowledge base, and processes the data returned by the perception system. When a user queries the knowledge base for a specific object class, “comp_object_detect” initiates the object detection and recognition request to the perception system. Then, the knowledge query and reasoning system filters the commonsense knowledge based on data returned by the perception system, identifying object instances that match the query or its subclasses. The data returned by the perception system includes object category labels. The filtering process involves determining whether the label is defined as a class in the knowledge base, and it is the process of querying either the category itself or its subclasses. This filtering process can be implemented using the Prolog built-in predicate “rdfs_subclass_of.” Taking the query for fruits as an example, when the perception system detects and locates objects such as apples, bananas, and plates, by evaluating whether the return value of “rdfs_subclass_of(L, 'Fruit')” is true (where L represents the object label returned by the perception system, and “Fruit” represents the queried fruit category), it can be determined that apples and bananas belong to the fruit category, thereby creating and returning instances of apples and bananas. Finally, all object instances belonging to the queried class are returned to the user. The synchronous communication pipeline is shown in Figure 6. Meanwhile, in the asynchronous communication mode, the perception algorithm is encapsulated as a ROS publisher node, and object instance generation is implemented by calling a custom Prolog predicate in subscriber nodes. “object_detect_listener” monitors sensory data from the perception system, detecting and generating object instances at regular intervals. The knowledge representation of object instances includes ID, class, timestamp, size, and pose, which can be extended according to task requirements and perception algorithms. Figure 7 displays the partial knowledge topology of “Instance1” of “Class1.” The “latestDetection” indicates the most recent detection results.

**Figure 8:**
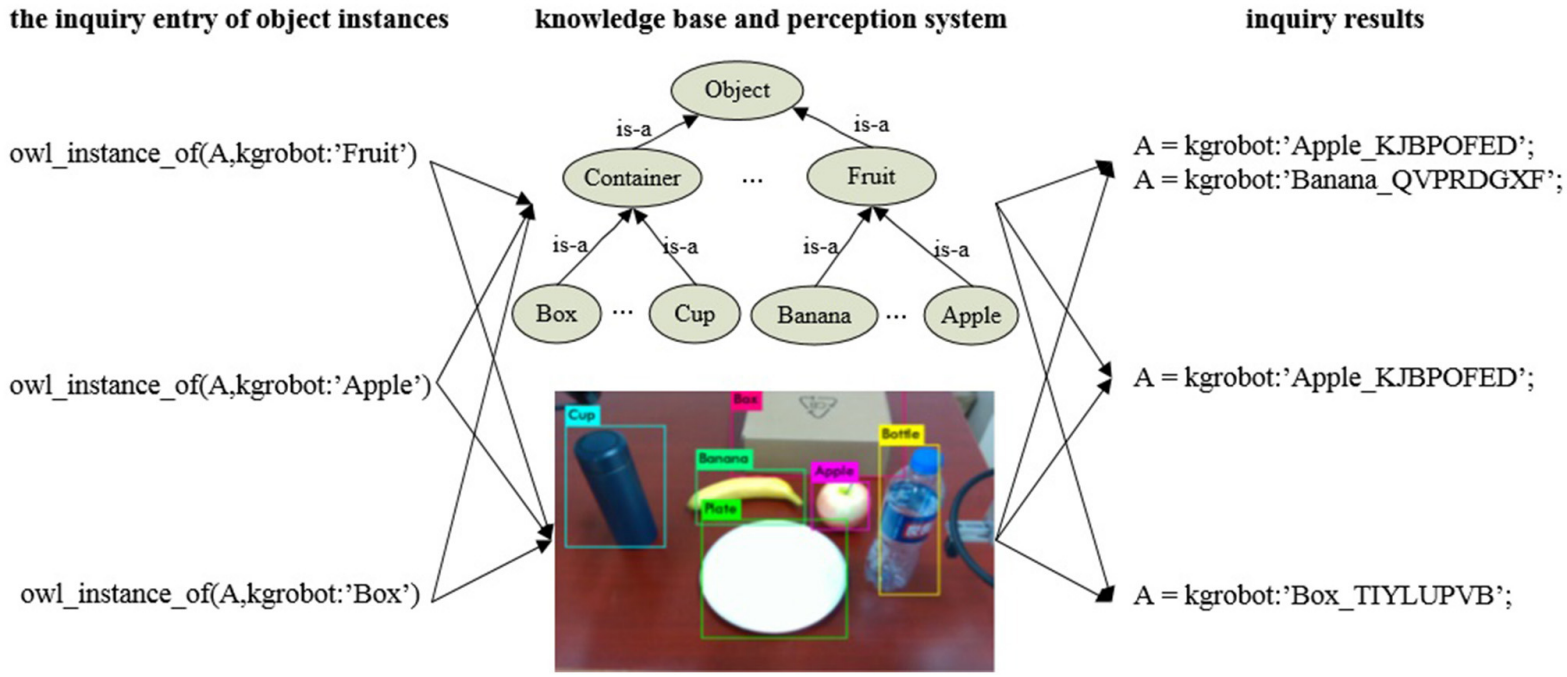
Object instance inquiry based on the knowledge base and perception system. Through the query interface, we can not only obtain instances of labeled objects in the perception system but also acquire instances of abstract concepts (such as “fruit“).

We choose YOLO v3 for object detection, which strikes a good balance between accuracy and efficiency. Here, Yolo v3 can be replaced with any object detection algorithm. In Figure 8, three types of objects, namely apple, banana and box, are queried and recognized. It can be seen from Figure 8 that there is information exchanged between the knowledge base and the perception system, which can be extended to multi-class objects or objects of different abstraction levels. Deep learning-based object detection and recognition approaches improve the generalization and scalability of ARTProF for object perception. Consequently, the robot is able to associate the physical objects detected in the environment with the abstracted knowledge about object classes.

## 4 Knowledge-guided manipulation operators

### 4.1 Action knowledge representation

Action knowledge is used to describe the actions executed by robots in manipulation tasks. In ARTProF, the relationships between the actions and objects are defined in the constraint attributes {*preActors, postActors*}, where *preActors* are the pre-conditions necessary for the action, specifying the action properties that are needed to be satisfied before the action is executed. *postActors* define the post-effects of the action, describing the environment's state after successful execution of the action.

**Figure 9:**
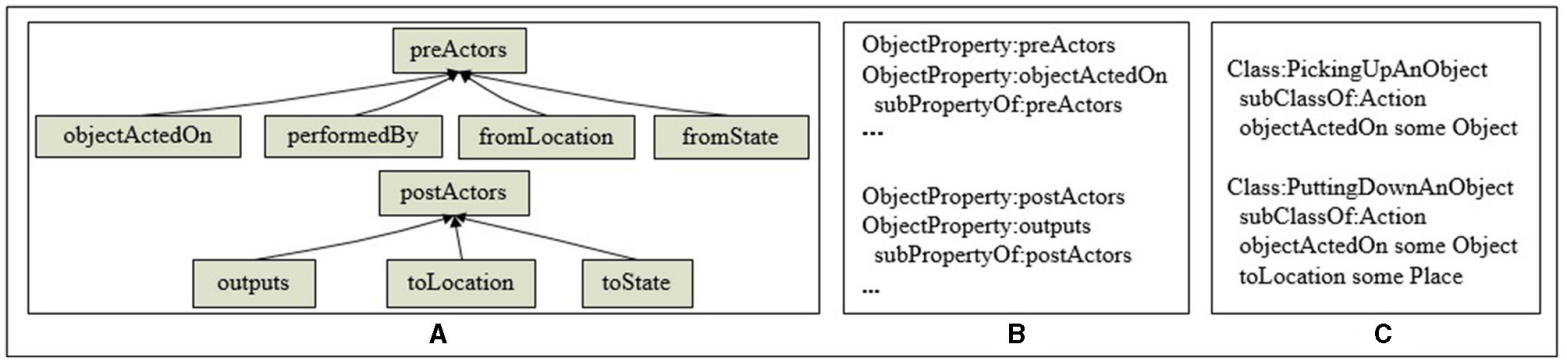
Action knowledge representation. **(A)** The hierarchy of the action attributes. **(B)** The definition of the action attributes. **(C)** An example of the knowledge representation of the action.

As shown in Figure 9A, *preActors* include: {*objectActedOn*, *performedBy*, *fromLocation*, *fromState*}, representing the operated object, the action execution agent, initial position and the initial state of the operated object, respectively. The *postActors* include: {*outputs*, *toLocation*, *toState*}, representing the output object, target position and target state of the operated object, respectively. The action attribute is defined using OWL pseudo-code, as shown in Figure 9B. Figure 9C showcases the OWL pseudo-code of two basic actions: picking up and putting down.

### 4.2 Manipulation operator

In ARTProF, manipulation operators based on ROS are designed to guide robot actions via knowledge. The integration with ROS is to maximize the utilization of existing robot operation algorithms. These operators are instances of the “Algorithm” class, which is a subclass of “Math” in the ontological knowledge base. The constraints for actions and objects are respectively fulfilled by “operatorAction” and “objectActedOn.” Basic operation algorithms on ROS are associated with ROS-related properties such as “serviceName,” “serviceReq,” “serviceRes,” and “serviceSrv.”

**Figure 10:**
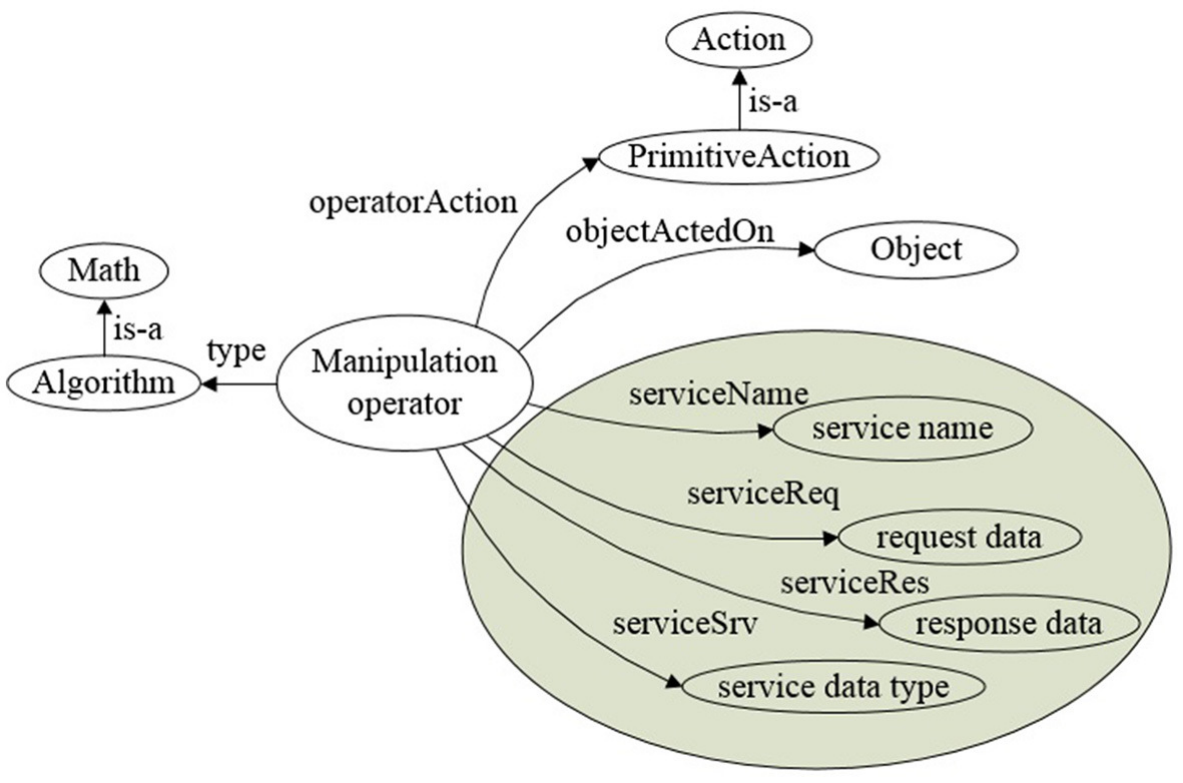
Manipulation operator. Manipulation operator is defined as instances of the “Algorithm” class in OWL, guiding robot actions based on ROS.

More specifically, as shown in Figure 10, the “PrimitiveAction” denotes the primitive action class in the knowledge representation. The “Object” corresponds to the “Object” class in the knowledge base, which describes the category of the operated object and its related semantic attributes. “service name”, “request data,” “response data,” and “service data type” are the service name, function name for obtaining the requested data, response data name, and service data type defined in ROS, respectively.

For example, in the task of “grasp an apple,” a manipulation operator named “PickingUpAnApple” is defined in ARTProF. The OWL pseudo-code of this operator is described as:


Individual: PickingUpAnApple
  type: Algorithm
  operatorAction: PickingUpAnObject
  objectActedOn: Apple
  serviceName: “pickingUpApple”
  serviceReq: “pickingUpAppleReq”
  serviceRes: “status”
  serviceSrv: “pickUpApple”
 
ObjectProperty: operatorAction
  domain: Algorithm
  rang: Action
 
DatatypeProperty: serviceName
  domain: Algorithm
  rang: string
...


During task execution, when the robot is performing the task, it first queries the knowledge base using the primitive action “PickingUpAnObject” and the operated object “Apple” to determine the manipulation operator “PickingUpAnApple.” The “grasp” operation is then invoked through ROS-related attributes of the manipulation operator to finish the task. The process of searching and reasoning for the manipulation operator is represented by Prolog pseudo-codeas follows:


get_operator(Action, Object, Operator) :-
  rdf(Operator, type, Algorithm),
  rdf(Operator, operatorAction, Action),
  rdf(Operator, objectActedOn, Object).


If the “Action” (the primitive action) and the “Object” (the operated object) are determined, the manipulation operator “PickingUpAnApple” is obtained through the “get_operator” operator.

### 4.3 Object operation similarity model

In human experiences, objects with similar characteristics can be manipulated in a similar manner. For example, apples and oranges, or water bottles and milk bottles can be manipulated upon using the same manipulation operator. To endow the robot with similar flexibility, ARTProF introduces an object operation similarity model. This model determines suitable manipulation operator for an unknown object based on object similarity. The object similarity is defined as:


(1) $$\begin{array}{l} \begin{array}{c} SIM\left(a_{1},a_{2}\right)=Sig\left(\overset{N}{\underset{i=1}{\sum }}S_{i}\right),\;a_{1},a_{2}\in A\\ Sig\left(x\right)=\frac{1}{1+exp\left(-k*x\right)} \end{array} \end{array}$$

where *SIM* denotes the object similarity with values ranging from [0, 1]. Higher values indicate larger object similarity. *a*_1_ and *a*_2_ are two object instances being compared. *A* is the set of object instances. *N* is the number of object features. *S*_*i*_ represents the similarity of objects based on feature *i*. *k* is used to adjust the slope of the sigmoid function. Decreasing the value of *k* will slow down the speed of approaching the limiting values.

The object similarity model extracts object features that directly influence the robot's manipulation of the object. The similarity of object features is then computed. The object features include: {category, shape, material, and size}. The influences of different features on the object similarity is manually defined. Table 1 shows object similarities with the same, different or unknown conditions of category, shape, and material. Table 2 shows object similarities of different sizes, measured using deviation intervals *d*. The volume deviation uses a unit of 10*cm*^3^, while length, width, and height deviations use units of 2*cm*, 1*cm*, and 1*cm*, respectively. The object similarities are shown in Table 3 [*k* is set to 0.3 in Equation (1)]. The results align with human cognition.

**Table 1:** Category, shape, and material feature similarity measure.

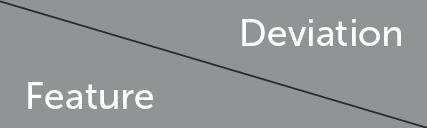	**Same**	**Different**	**Unknown**
Category	10	0	0
Shape	4	-4	0
Material	1	-1	0

**Table 2:** Size feature similarity measure.

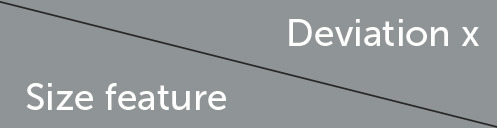	**|*x*| ≤ 1*d***	**1*d* < |*x*| ≤ 2*d***	**2*d* < |*x*| ≤ 3*d***	**3*d* < |*x*| ≤ 5*d***	**|*x*|>5*d***
Volume (cm^3^)	1	0	0	0	-1
Length (cm)	1	0	0	-1	-1
Width (cm)	2	0	-1	-2	-2
Height (cm)	1	0	0	-1	-1

**Table 3:** Example of object similarity.

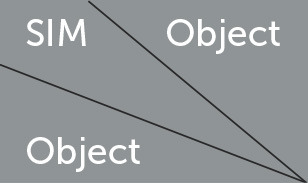	**Apple**	**Orange**	**Water bottle**	**Milk bottle**
Apple	1.0	0.95	0.14	0.23
Orange	0.95	1.0	0.14	0.23
Water bottle	0.14	0.14	1.0	0.77
Milk bottle	0.23	0.23	0.77	1.0

The category and size features can be obtained in real-time through perceptual algorithms such as YOLO v3 and SSD. The shape and material are static features defined directly in the knowledge base through attributes “shapeOfObject” and “materialOfObject.” The manipulation operator is then searched based on the object operation similarity model. All manipulation operators associated with the basic action are identified. The similarity between the current operated object and the object in each manipulation operator is calculated. Finally, the manipulation operator with the highest similarity is selected, and the corresponding action is executed.

## 5 Dynamic task planning

### 5.1 Task knowledge representation

A robotic task can often be decomposed into several low-level primitive actions. The aim of the task planning is to rearrange the primitive actions with preconditions and effects to achieve the task. The knowledge representation in ARTProF adopts a hierarchical structure. The OWL pseudo-code for retrieving an apple is described as:


Class: TakeAnAppleToPlate
  subClassOf: PuttingSomethingSomewhere
  objectActedOn some Apple
  toLocation in Plate
 
Class: PuttingSomethingSomewhere
  subClassOf: Action
  subAction some PickingUpAnObject
  subAction some PuttingDownAnObject
 
Class: PickingUpAnObject
  subClassOf: Action
  objectActedOn some Object
 
Class: PuttingDownAnObject
  subClassOf: Action
  objectActedOn some Object
  toLocation some Place
...


The task of “TakeAnAppleToPlate” is defined as a subclass of “PuttingSomethingSomewhere.” The predicates “objectActedOn” and “toLocation” are used to define the operated object and its target location. The “PuttingSomethingSomewhere” class as a subclass of “Action” comprises sub-actions “PickingUpAnObject” and “PuttingDownAnObject.” The sub-action “PickingUpAnObject,” as a subclass of “Action,” is constrained by the predicate “objectActedOn.” The sub-action “PuttingDownAnObject,” which is also a subclass of “Action,” is constrained by the predicates “objectActedOn” and “toLocation.”

### 5.2 Task execution

Task knowledge guides the process of robotic task execution. Upon receiving a task execution command, the robot initiates the task planning process by inquiring the task knowledge from the knowledge base to create the task instance. The action execution sequence is then obtained. The action instances are generated following the constraints of the task (e.g., preconditions and effects). Both task instances and action instances are stored in memory as temporary knowledge using a prolog-based representation. The pseudo-code of the generated task and action example of retrieving an apple is described as:


Instance: TakeAnAppleToPlate_001
  type: TakeAnAppleToPlate
  ObjectActedOn: Apple
  toLocation: in Plate
  subAction : PickingUpAnObject_001
  subAction : PuttingDownAnObject_001
 
Instance: PickingUpAnObject_001
  type: PickingUpAnObject
  objectActedOn: Apple
 
Instance: PuttingDownAnObject_001
  type: PuttingDownAnObject
  objectActedOn: Apple
  toLocation: in Plate
...


The generated task instance “TakeAnAppleToPlate_001” includes two action instances “PickingUpAnObject_001” and “PuttingDownAnObject_001.” The action instance “PickingUpAnObject_001” is subject to task constraints. The operated object is replaced from the abstract “Object” by the specific object “Apple.” Similarly, the abstract position “Some Place” is replaced by “in Plate” in “PuttingDownAnObject_001.”

### 5.3 Dynamic task planning

In ARTProF, we introduce a dynamic task planning method called Action Primitive Conditional Exploration Dynamic Task Planning. The robot performs each primitive action based on its prior task knowledge. During task execution, if a primitive action fails to meet the execution preconditions. the system initiates a search for a new action sequence through knowledge reasoning. This process continues until a valid primitive action, meeting the execution preconditions, is successfully executed. The task is considered completed once all defined primitive actions have been carried out.

Suppose the task is denoted by *T*, and the action is denoted by *a*. The task can be represented as *T* = [*a*_1_, *a*_2_, *a*_*i*_, …, *a*_*n*_], where *a*_*i*_ represents the *i*-th primitive action in the action sequence. This task comprises an ordered composition of *n* primitive actions. The description of the algorithm is shown in Algorithm 1.

In Algorithm 1, *T*′ denotes the set of actions generated, initialized to ∅. *len* is the action depth, initialized to 0. When an action fails to meet the execution condition, the action depth is increased by 1, and this process iterates. The maximum action depth is denoted by *MAXLEN*. *PS* is the set of action sequences generated through knowledge reasoning represented by *getPreActionsList*. *P* denotes the action sequence that meets the execution conditions for the current action. The function *actionExe*(*a*) denotes the robot's execution of action *a*. By default, if action *a* fails during execution and still meets the execution conditions, it will be executed again until successful.

**Figure 11:**
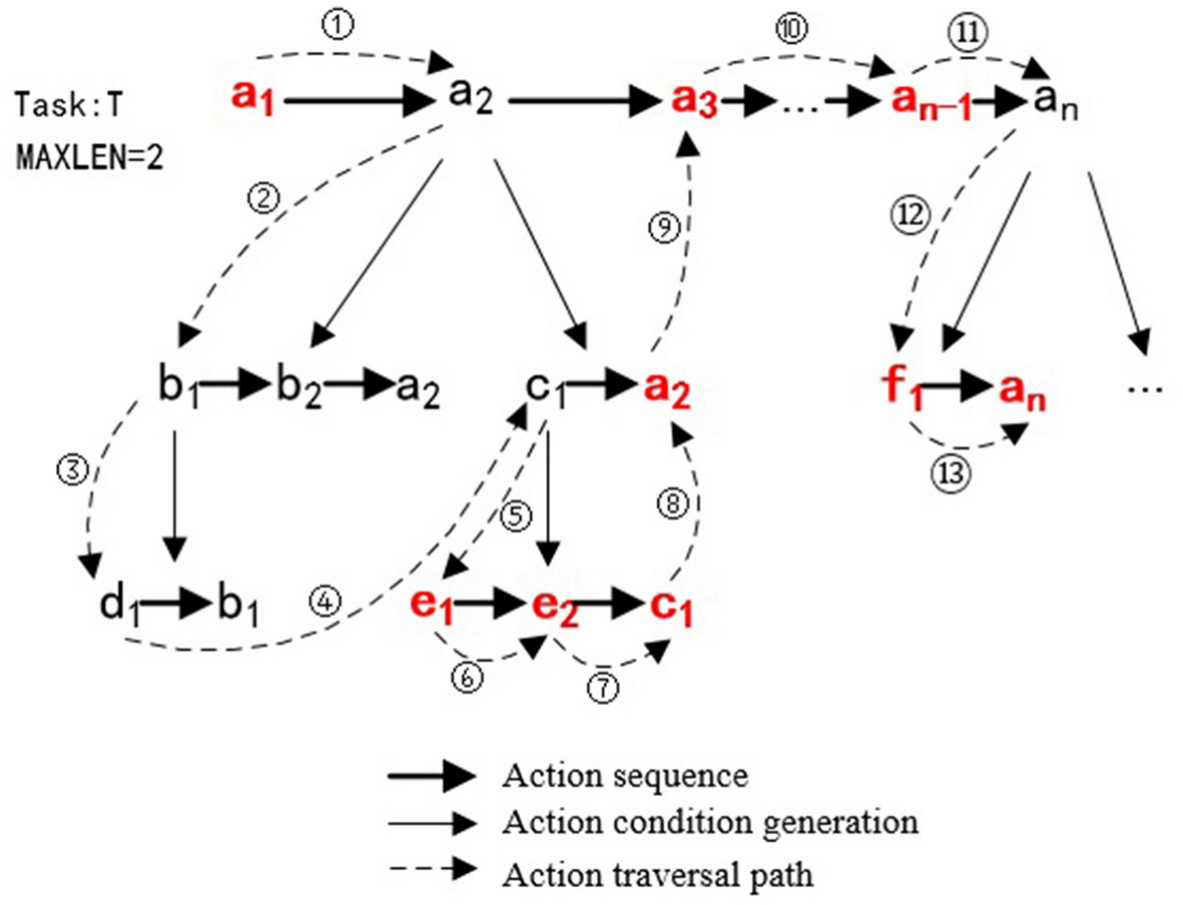
The execution process of the dynamic task planning. The initial action sequence for the task is [*a*_1_, *a*_2_, *a*_3_, ..., *a*_*n*−1_*a*_*n*_], and during the task execution process, dynamic planning is triggered due to some actions not satisfying the execution conditions. The actual execution action sequence becomes [*a*_1_, *e*_1_, *e*_2_, *c*_1_, *a*_2_, *a*_3_, ..., *a*_*n*−1_, *f*_1_, *a*_*n*_].

Assuming task *T* = [*a*_1_, *a*_2_, *a*_*i*_, ..., *a*_*n*_], the execution process of the dynamic task planning algorithm is shown in Figure 11. Initially, the system executes *a*_1_ and plans *a*_2_ next. As *a*_2_ fails the execution conditions, the system explores alternative action sequences [*b*_1_, *b*_2_, *a*_2_] and [*c*_1_, *a*_2_] through knowledge reasoning. After checking that *b*_1_ does not meet the execution conditions, and the action depth of *d*_1_ is already at the maximum depth (*MAXLEN*=2), the system backtracks to execute [*c*_1_, *a*_2_]. Upon evaluation, [*e*_1_, *e*_2_, *c*_1_] are successfully executed, and the system implements *a*_2_, *a*_3_, etc.

The dynamic task planning relies on task ontological knowledge as the prior knowledge. Throughout the execution of robotic tasks, new actions are explored by considering both depth and breadth. This strategy empowers the robot with adaptability to work in unstructured environments while avoiding the shortcomings of manually editing domain knowledge in traditional task planners.

## 6 Experimental results and analysis

### 6.1 Experiment 1: autonomous retrieval of manipulation operators with objects in the scene

**Figure 12:**
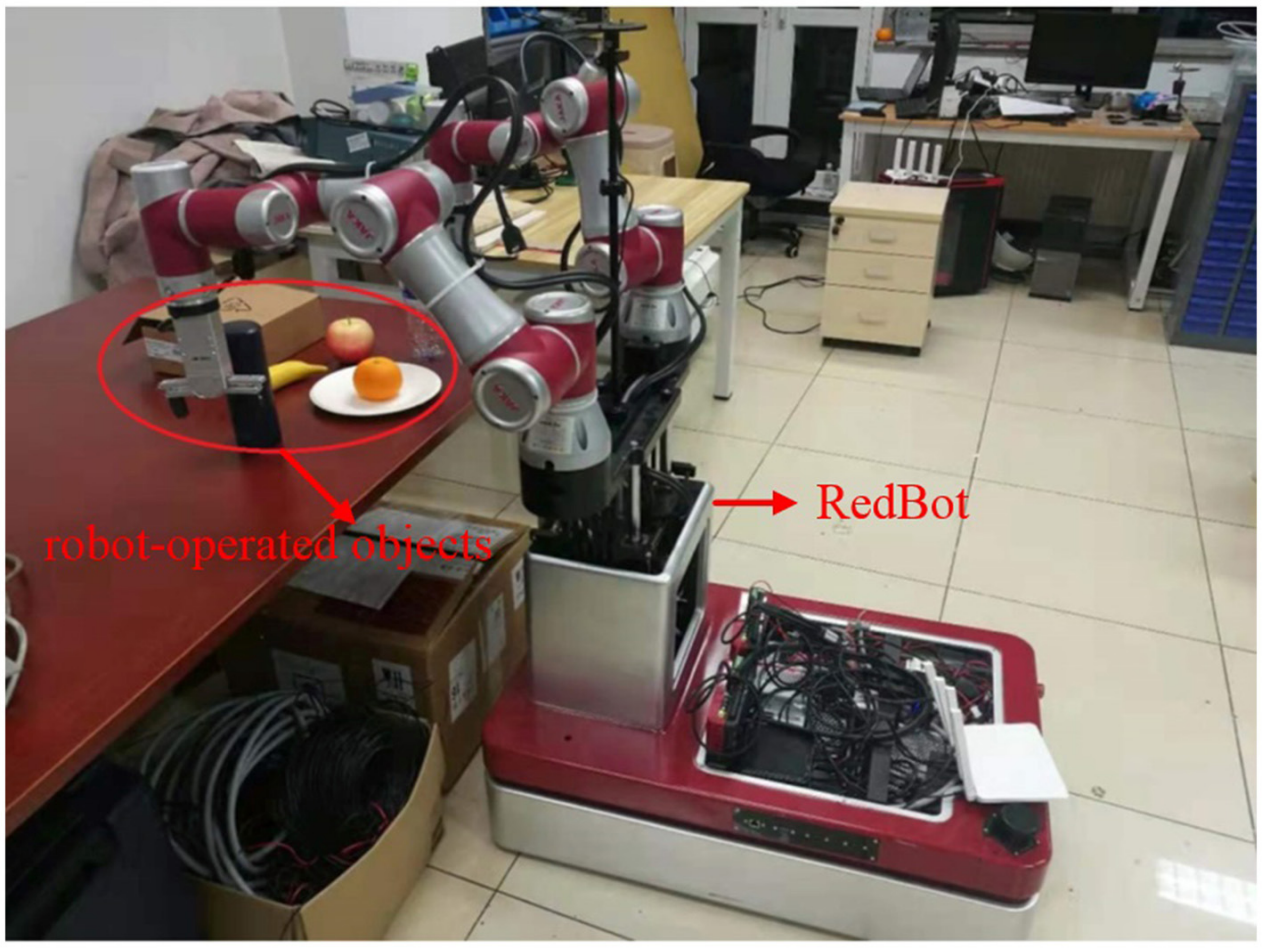
Experimental environment for autonomous retrieval of manipulation operators with objects.

The experiments are carried out in a laboratory environment shown in Figure 12. The robotic system, named RedBot, is equipped with two 6-degrees-of-freedom (DOF) manipulators, a mobile platform, two single line radars, an industrial computer, and an RGB-D camera (Intel RealSense D435i). The software modules include mapping, navigation, obstacle avoidance, object detection and recognition, grasping, etc. These modules are communicated through ROS messages. The robot is controlled by a back-end server. The back-end platform utilizes containers to isolate distinct software environments. The data exchange between the robot and the back-end platform is facilitated through Ethernet.

The robot demonstrates capability in grasping various objects such as oranges, cups, and footballs, etc, as indicated in Table 4. Corresponding manipulation operators for these grasp actions are denoted as “pickingUpOrange,” “pickingUpCup,” and “pickingUpFootball.” However, real-world scenarios often introduce novel objects to the robot. In our experiments, we also conduct grasping experiments involving apples, water bottles, and watermelons, of which the manipulation operators are not specified in the knowledge base.

**Table 4:** Features of objects to be grasped.

**Object**	**Category**	**Shape**	**Material**	**Volume (*cm*^3^)**	**Length (*cm*)**	**Width (*cm*)**	**Height (*cm*)**	**Manipulation operator**
Orange	Orange	Sphere	Fruit	512	8	8	8	pickingUpOrange
Cup	Cup	Cylinder	Metal	540	6	6	15	pickingUpCup
Football	Football	Sphere	Leather	8,000	20	20	20	pickingUpFootball
Apple	Apple	Sphere	Fruit	512	8	8	8	pickingUpOrange
Waterbottle	Bottle	Cylinder	Plastic	720	6	6	20	pickingUpCup
Watermelon	Watermelon	Sphere	Fruit	8,000	20	20	20	pickingUpFootball

**Figure 13:**
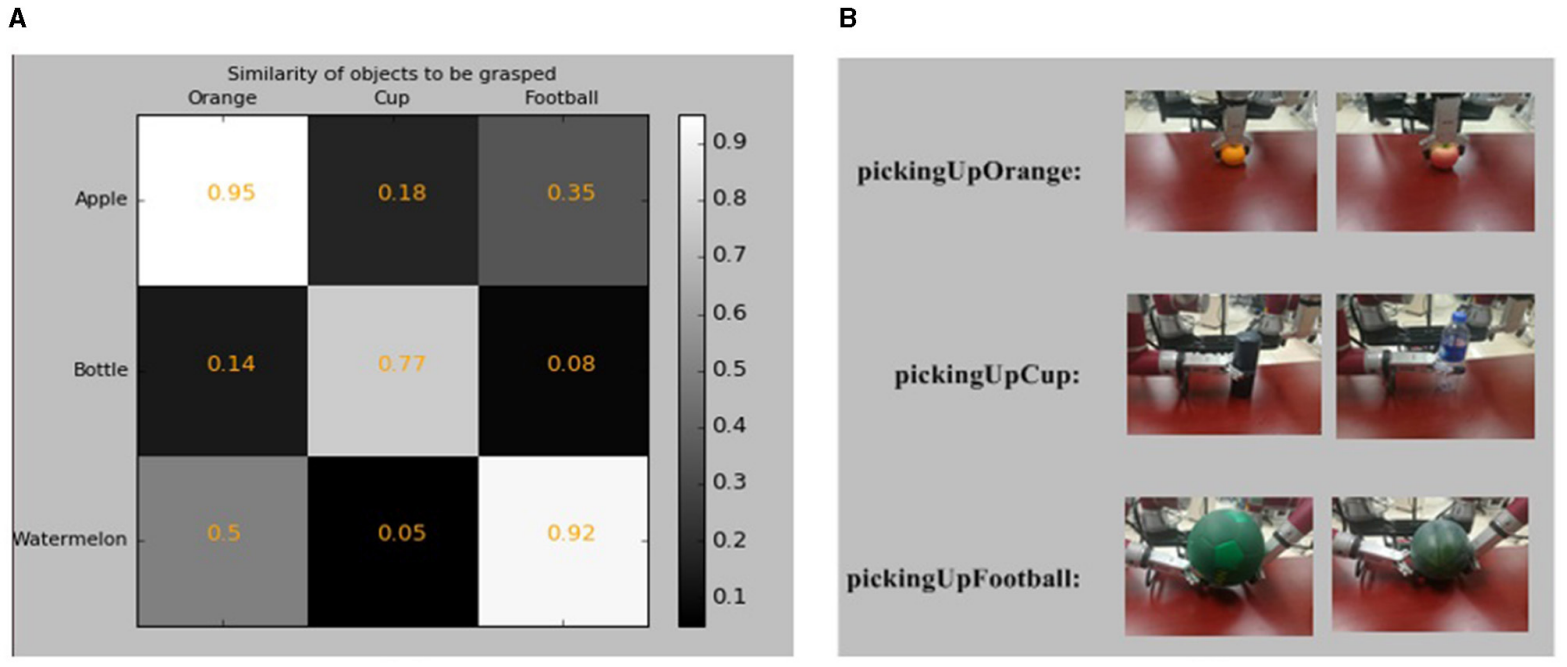
The experimental results of operation transfer. **(A)** The Similarities of objects to be grasped. Darker cells indicate lower similarities between objects. **(B)** The robot grasping of different objects and the corresponding manipulation operators. The grabbing of apples and oranges uses the same manipulation operator “pickingUpOrange.” Similarly, the grabbing of the cup and water bottle uses the same manipulation operator “PickingUpCup,” and the grabbing of the football and watermelon uses the same manipulation operator “PickingUpFootball”.

In our grasping experiment, the robot is placed in front of a table where the objects are placed. As there is no manipulation operator for grasping apples, water bottles, and watermelons in our knowledge base, the robot selects the manipulation operators by evaluating the similarities between objects. The object's shape and material are obtained from the knowledge base as prior knowledge. The perception system of ARTProF provides measurements for the length, width, and height of the objects, from which the volume is calculated as *V* = *L*×*W*×*H*. The similarity between objects is calculated as shown in Figure 13A. Darker boxes indicate lower similarity. The robot grasping of different objects and the corresponding manipulation operators are illustrated in Figure 13B. The experiments highlight the robot's ability not only to autonomously match manipulation operators for objects in the scene but also to generalize operators across different objects.

### 6.2 Experiment 2: dynamic task planning

**Figure 14:**
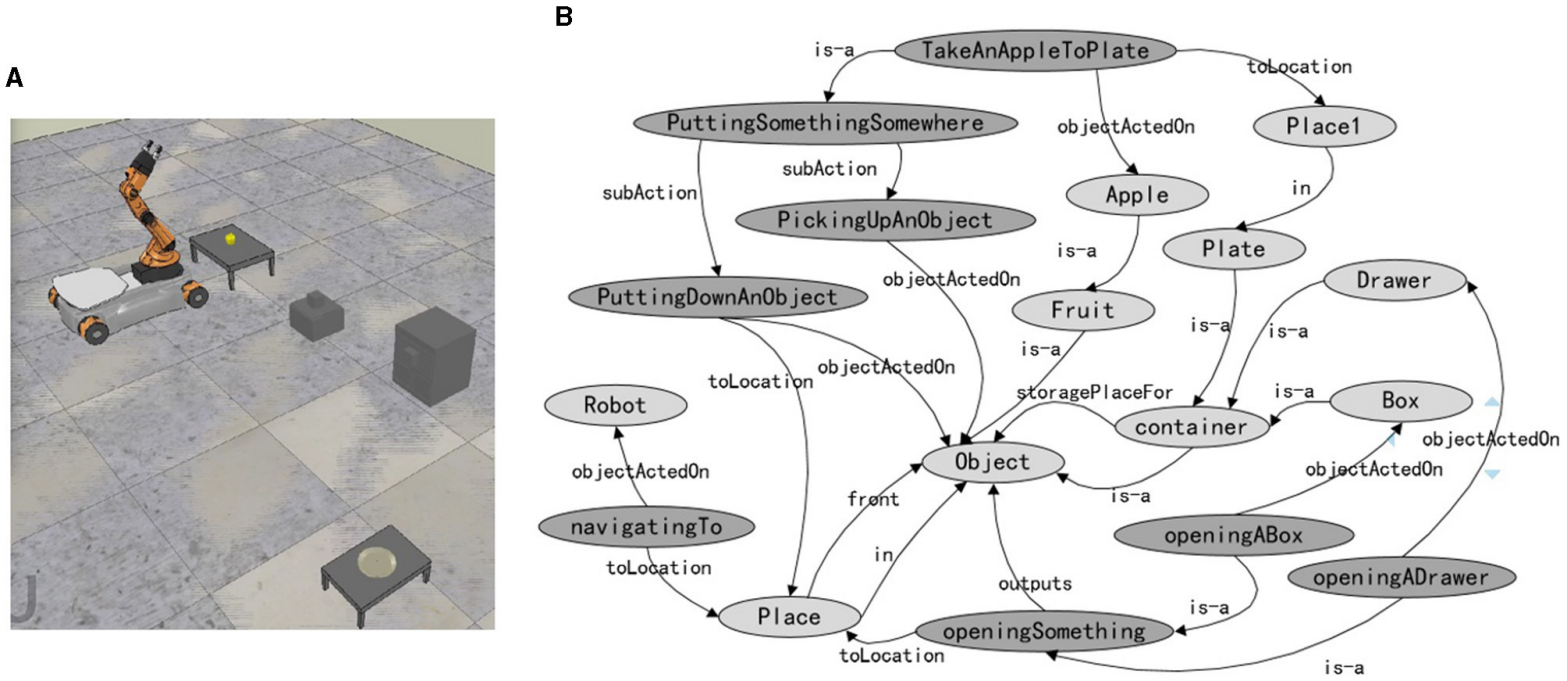
The scenario and the background knowledge of the dynamic task planning. **(A)** The scenario of the dynamic task planning. The location of the apple is uncertain, it could be placed on the table near the robot or in a box or drawer far from the robot. **(B)** The semantic map of the task-related background knowledge. The dark nodes are task knowledge, and the light nodes are environmental knowledge.

**Figure 15:**
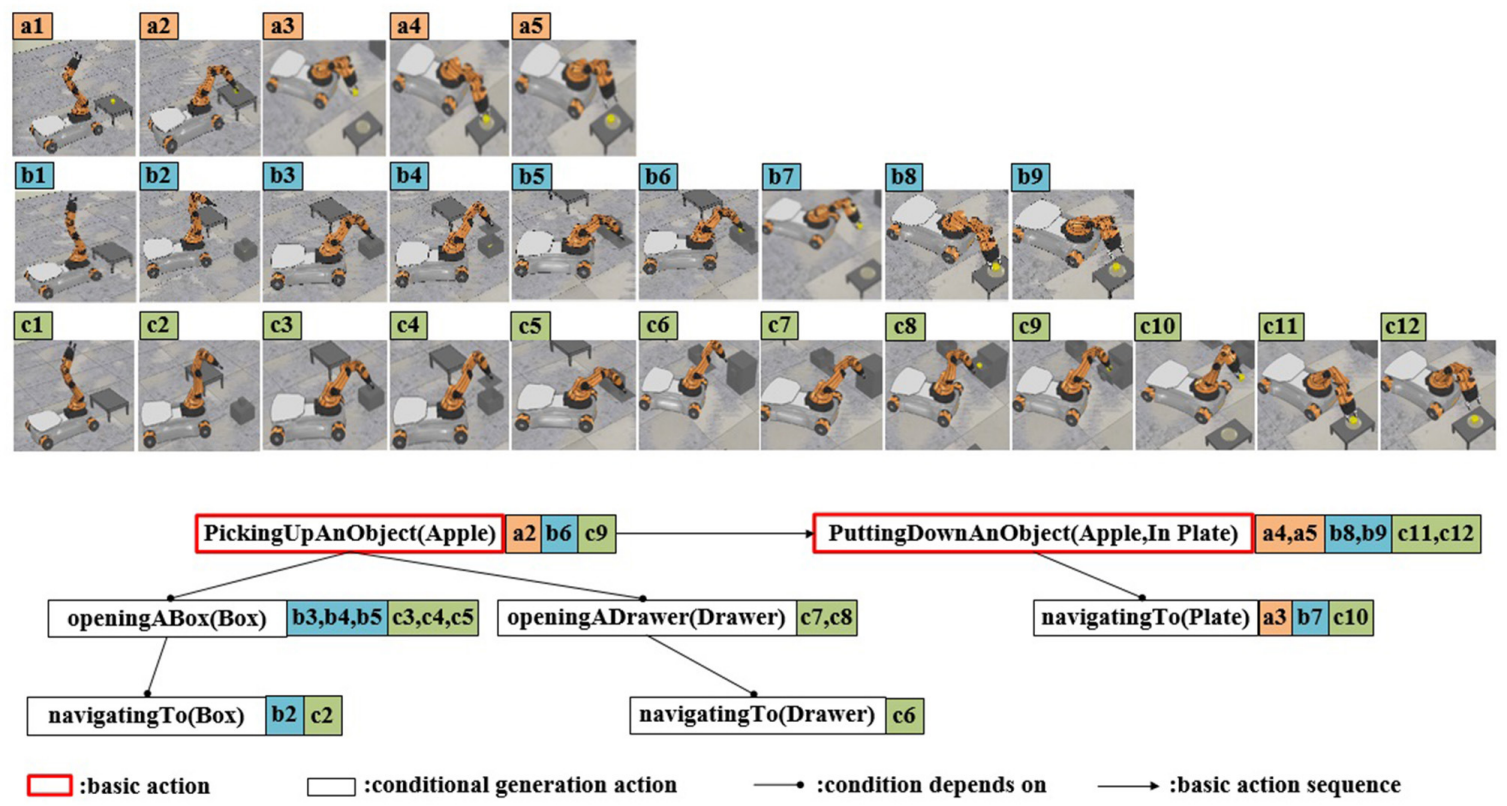
Screenshots of robot's dynamic task planning experiment. **(a1–a5)** The apple is placed on the table, and there are no instances of action execution failure. **(b1–b7)** The apple is placed on the table, and the first attempt at the “PickingUpAnObject” action fails. **(c1–c9)** The apple is in the box. **(d1–d12)** The apple is in the drawer.

This experiment takes retrieving an apple as an example to achieve task planning in unknown environments. The dynamic task planning experiment is conducted in the simulation platform CoppeliaSim (version 4.1.0). The indoor experimental setting, as shown in Figure 14A, includes a YouBot by KUKA, an apple, a plate, a box, a cabinet with drawers, and two tables. The semantic map of the task-related background knowledge is shown in Figure 14B, where the dark nodes are task knowledge, and the light nodes are environmental knowledge. The grasping task is defined as two actions: picking up and putting down. The knowledge representation of the task is presented in Section 5.1. In scene 1 and scene 2, the apple is placed on the table, as shown in Figures 15a1, b1. In scene 3, the apple is in the box, as shown in Figure 15c1. In scene 4, the apple is in the drawer, as shown in Figure 15d1.

In scene 1, the “pick-up” action is executed successfully. However, the subsequent “put-down” action fails due to the “toLocation” (plate location) is beyond the robot arm's operational range. According to the dynamic task planning, the “navigatingTo” action is triggered. Once the navigation is completed, fulfilling the conditions for the “put-down” action, the “put-down” action is executed accordingly. The final action sequence involves picking up the apple → navigating to the plate → putting down the apple, as illustrated in Figures 15a2–a5.

In scene 2, during the execution of the “pick-up” action, the apple falls, resulting in the failure of the action execution. However, since the conditions for executing the “pick-up” action are still met at this point, the “pick-up” action is repeated. After successfully execution of the pick-up action, the subsequent task execution process follows the same pattern as in scene 1. The final action execution sequence involves picking up the apple (performed twice) → navigating to the plate → putting down the apple, as illustrated in Figures 15b2–b7. In scene 3, the robot fails to perceive the apple, leading to the unmet condition for the “pick-up” action. After the knowledge reasoning in ARTProF, the robot infers the potential presence of the apple within the box. Consequently, it performs the “openingABox” operation. Once the apple is found, the subsequent actions are similar with those of scene 1, resulting in the final action sequence: navigating to the box → opening the box → picking up the apple → navigating to the plate → putting down the apple, as shown in Figures 15c2–c9.

In scene 4, the apple is not present in the box. According to dynamic task planning, the robot infers the potential presence of the apple within the drawer. Since the drawer is not within the operating range of the manipulator, the robot navigates to the drawer firstly. The final action sequence of the robot is: navigating to the box → opening the box → navigating to the drawer → opening the drawer → picking up the apple → navigating to the plate → putting down the apple. The execution process is shown in Figures 15d2–d12. Experimental details are demonstrated in the Supplementary video.^1^ Experimental results show that the effectiveness of the proposed ARTProF in improving the robot's adaptability within unstructured and dynamic environments.

## 7 Conclusions

In this paper, we proposed an Ontology based Autonomous Robot Task Processing Framework (ARTProF) to improve the robot's adaptability within unstructured and dynamic environments. ARTProF includes key functionalities such as knowledge representation, knowledge reasoning, and task planning and control. The interface between the knowledge base and the neural network-based object detection algorithms augments the perception capabilities of the robots. To bridge the gap between the knowledge base and robot actions, the framework defines ROS based manipulation operators. ARTProF also introduces an operation similarity model, enabling the robot to generalize operations to novel objects effectively. A dynamic task planning method based on knowledge reasoning is further proposed for autonomous task planning. Experimental results showcase improvements of ARTProF in the robot's environmental perception, generalization abilities, and autonomous task execution within unstructured and dynamic environments. Ongoing research is focused on refining the ARTProF framework by integrating neurosymbolic inference.

## Data availability statement

The original contributions presented in the study are included in the article/supplementary material, further inquiries can be directed to the corresponding authors.

## Author contributions

YG: Writing – original draft. SZ: Validation, Writing – review & editing. YC: Writing – review & editing. TL: Writing – review & editing. HW: Validation, Writing – review & editing. XH: Validation, Writing – review & editing. SW: Conceptualization, Funding acquisition, Methodology, Writing – review & editing.

## Footnotes

^1^ https://doi.org/10.6084/m9.figshare.25531045.v2
